# Current trends in the use of psychodrama and drama therapy in the treatment of mental disorders

**DOI:** 10.1192/j.eurpsy.2022.1929

**Published:** 2022-09-01

**Authors:** M. Ciołek, K. Kamińska, M. Nowak, K. Krysta

**Affiliations:** 1 Medical University of Silesia, Students` Scientific Association, Department Of Rehabilitation Psychiatry, Katowice, Poland; 2 Medical University of Silesia, 2. Department Of Biophysics And Student Science Club Named Prof. Zbigniew Religa, Zabrze, Poland; 3 Medical University of Silesia, Department Of Rehabilitation Psychiatry, Katowice, Poland

**Keywords:** Mental Disorders, psychodrama, drama therapy

## Abstract

**Introduction:**

Psychodrama and Drama therapy enable patients to establish contact with the threat of stepping into a given role. This gives the opportunity to learn how to control it, which leads to better expression of oneself and better communication with the environment. Those qualities are crucial in the treatment of mental disorders. Despite the variety of literature describing the methodology, clinical trials using these forms of therapy are relatively rare.

**Objectives:**

To describe the current trends in psychodrama (PD) and drama (DT) research over the last 6 years.

**Methods:**

We have implemented a systematic approach to literature review, consistent with the PRISMA declaration. We searched through major medical databases: PubMed, Web of Science, Scopus by Elsevier and Science Direct for peer-reviewed articles published between 2015 and 2020. We have included studies using all types of methodology: mixed, quantitative and qualitative and also case studies. The risk of bias was assessed for randomized clinical trials, consistent with the PRISMA declaration.

**Results:**

Using our search strategy we have identified 24 publications with 454 participants. Most of the subjects were adults, only four studies focused on children. Overall, these studies looked at the effects of PD i DT on more than 25 different outcomes. Therefore theatre - based therapies research reports promising results across all methodologies. Although, most of the interventions have small groups of clients and are not randomized.

**Conclusions:**

Current reports on the effectiveness of PD and DT still need to be verified on a larger group of patients.

**Disclosure:**

No significant relationships.

